# Phylogenomic analysis supports the deep and gradual evolution of water-conducting tissues

**DOI:** 10.1093/plphys/kiag525

**Published:** 2026-07-18

**Authors:** Alexander M C Bowles

**Affiliations:** Department of Biology, University of Oxford, Life and Mind Building, South Parks Road, Oxford OX1 3EL, United Kingdom

## Abstract

The origin of land plants was marked by the evolution of key adaptations to terrestrial environments. The patchy distribution of many of these traits in bryophytes, including water-conducting tissues, raises questions about whether these features share a common evolutionary origin or arose independently. Molecular data from 160 species were analyzed using phylogenomic approaches to investigate bryophyte genome evolution and the origins of water-conducting tissues. Hornworts were identified as the most genomically reduced bryophyte lineage. Many of the gene families absent in hornworts are associated with developmental processes, including those important for water-conducting cells, which are not found in this group. Comparative analyses of xylem developmental gene networks across bryophytes revealed a mosaic of evolutionary mechanisms—gene loss, duplication, and co-option—underpinning the emergence of water conduction. These results indicate that reductive, convergent, and tracheophyte-specific evolutionary processes all contributed to the development of water-conducting tissues in land plants. Together, these findings highlight how multiple genetic mechanisms drove the deep and gradual evolution of water-conducting tissues, a key innovation that enabled the successful establishment of plants in terrestrial environments.

## Introduction

Around 5 hundred million years ago, the first plants moved from aquatic environments onto land, transforming the atmosphere and promoting the evolution of animals and fungi (Morris et al. 2018; Bowles et al. 2023). Subsequently, an immense diversity of plant life evolved, resulting in the “emerald planet” of today (Beerling 2007).

The evolutionary relationships of land plants are improving (Puttick et al. 2018; Harris et al. 2020), a major barrier to investigating the characteristics of ancestral plants. Puttick et al. (2018) recovered the setaphytes, the liverworts and mosses, as a monophyletic clade, but they were unable to rule out paraphyletic bryophytes. They rejected all but 3 phylogenetic hypotheses, each differing in the placement of hornworts, a lineage central to the present study. Subsequently, Harris et al. (2020, 2022) identified statistical support for the internal relationships of land plants based on analysis of phylogenomic data. Specifically, these analyses determined that bryophytes form a monophyletic group sister to vascular plants, with phylogenetic alternatives unequivocally rejected. Since, many others have reported these same relationships among the principal 4 land plant lineages (de Sousa et al. 2019; Leebens-Mack et al. 2019; Li et al. 2020; Su et al. 2021; Healey et al. 2023; Hu et al. 2023; Li et al. 2024; Bowles et al. 2024a, 2024b; Dong et al. 2025; Shen et al. 2025; Xue et al. 2026).

Despite this growing body of work supporting bryophyte monophyly, there are a set of papers that emphasize that establishing the root of the embryophyte crown group remains an area of active debate. Qiu and Mishler (2024), in their critique of recent phylogenomic studies, argued that the relationships among the 3 bryophyte lineages and vascular plants should still be regarded as unresolved. Importantly, the relationships among the 4 major embryophyte lineages represent a classic example that can lead to phylogenetic uncertainty. Each of the branches became differentiated hundreds of millions of years ago, most possibly because of a rapid radiation, followed by large-scale extinction events. Extant bryophytes therefore evolved relatively recently in geological time. This extinct diversity cannot be included in molecular-based phylogenetic analyses, leading to an extraordinarily long branch linking them to their closest extant relatives. Bell et al. (2020) explicitly attempted to understand the origin of such discrepancies, noting that the problem is most likely a rooting issue. It has historically been difficult to infer the root of land plants due to the long branch connecting them to streptophyte algae and the relatively short branches subtending major lineages of land plants, with different rooting strategies often implying different land plant relationships. To tackle this, Chen and de Vries (2025) investigate the effects of outgroup sampling on internal land plant relationships robustly showing that bryophyte monophyly remains despite outgroup sampling.

As these relationships are honed, our understanding of the evolution of genes, genomes, and traits of bryophytes and by extension, potentially the first land plants, has been strengthened. Recent insights have revealed that the first bryophytes underwent reductive evolution, indicating they descend from a more complex land plant ancestor (Harris et al. 2022). Vascular plants contrastingly have followed a different evolutionary path with genomes characterized by gradual gene gain and diversification. With unprecedented genomic sampling, bryophytes have also been shown to have a higher number of unique and lineage-specific gene families than their vascular plant relatives (Dong et al. 2025). This draws into question whether the patchy distribution of many important traits in bryophytes represents convergent evolution between bryophyte and vascular plant lineages or reductive evolution from the first land plants. Evidence from stomatal studies and hypothesized patterns in conductive tissues (xylem and phloem) suggest that reductive evolution may account for at least some of this trait distribution (Harris et al. 2020; Bowles et al. 2022).

**Figure 1:**
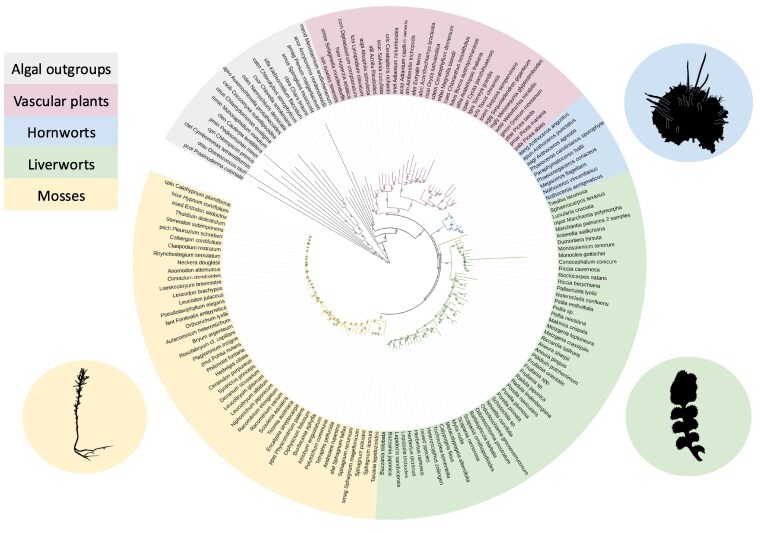
Phylogenetic tree of bryophytes based on 216 genes. Bootstraps highlight support at each node. This tree was fixed on the consensus topology from Harris et al. (2022). Silhouettes sourced from phylopic.org.

Since the transition of plants to land, 2 mechanisms of water transport have evolved: the water-conducting cells of bryophytes and the vascular tissue of vascular plants (Fig. 1) (Ohtani et al. 2017). Vascular tissue has traditionally been characterized as a novelty of vascular plants (Kenrick and Crane 1991; Ligrone et al. 2000; Edwards 2003) but recent studies have hinted to a more complex evolutionary history (Woudenberg et al. 2022). Physiological studies have shown strong parallels between the vascular function of the common haircap moss, *Polytrichum commune*, and that of vascular plants (Brodribb et al. 2020) while genetic studies revealed that vascular NAC transcription factors have equivalent functions in the development of water-conducting cells and xylem (Xu et al. 2014). Given these studies, a key unresolved question is whether water-conducting tissues in land plants evolved once, or on multiple occasions.

Here, genome evolution within bryophyte lineages and its relationship to water-conducting cell development is investigated. The monophyly of bryophytes is inferred based on analysis of 216 single-copy orthologs (Figure S1). Based on the inferences of a monophyletic group of bryophytes, comparative genomic analyses show that hornworts, the first splitting lineage of bryophytes and the group with no water-conducting species, have lost the largest number of genes. Functionally, these are associated with root development, disease resistance, and cellular organization. As well as gene loss, the complexities of bryophyte evolution could be explained by convergent genome evolution. Based on the inferences of a monophyletic group of bryophytes, gene tree–species tree analysis found that the bryophyte lineage most frequently associated with convergent gene family diversification with vascular plants were the hornworts. Further analysis of genetic pathways involved in xylem development revealed 3 classes of developmental genes: (i) genes diversifying in vascular plants; (ii) genes diversifying in parallel in vascular plants and water-conducting mosses; and (iii) genes absent in hornworts. To incorporate the uncertainties of bryophyte relationships, the results are interpreted under alternative phylogenetic frameworks, which support a model in which heterogeneous genetic mechanisms contributed to the morphological development of plant tissues. These point to the piecemeal composition of the genetic networks of water conduction, in a tracheophyte-specific manner, convergently between tracheophytes and bryophytes, and conserved across all water-conducting plants, respectively. This suggests that the water-conducting apparatus did not appear as a single complex innovation. Instead, it emerged through incremental genetic recruitment over deep evolutionary time, beginning before the divergence of extant land plant lineages.

## Methods

### Compiling a genomic dataset

Broad taxonomic sampling of genomic and transcriptomic data was implemented to accurately infer the origin and diversification of bryophyte gene content (Data S1). BUSCO analysis was used to assess the quality of genome annotation, using a threshold of <30% missing genes in the BUSCO Eukaryota dataset as a benchmark to accept data for further analysis (Data S1) (Waterhouse et al. 2018). In total, 160 viridiplantae taxa were downloaded equating to 4,202,729 predicted proteins, including 110 bryophytes, 31 vascular plants, and 19 Viridiplantae outgroup taxa.

### Orthology inference

OrthoFinder (v.2.3.7) was used to cluster protein coding genes into orthogroups (Emms and Kelly 2019), based on sequence divergence, using default settings (orthofinder -f data_folder). Orthofinder was launched on 31 January 2024, and therefore any genomes published after this date were not included in the analysis.

### Species tree analysis

Single-copy orthologs were identified using a previously described python script (Harris et al. 2020), which removes paralogous genes from orthogroups. The script enables the user to specify a minimum taxonomic occupancy of each orthogroup, set at 70% to select 216 genes. Single-copy orthologs were aligned using MAFFT (v.7.520) (Katoh and Standley 2013) using –auto parameter and trimmed with TRIMAL (v.1.4.1) (Capella-Gutiérrez et al. 2009) using the –automated1 parameter (Data S2). Multiple sequence alignments were concatenated using Phyutility (v.2.7.1) to create a supermatrix (Smith and Dunn 2008). A bootstrapped maximum likelihood phylogeny was inferred using IQ-Tree (v.2.2.3) (Nguyen et al. 2015) using the Bayesian Information Criterion (BIC) to select the best fitting substitution model and empirical profile mixture models (C10–C60). A total of 1,000 ultrafast bootstrap replicates were used. In IQ-TREE, this tree was fixed on the consensus topology from Harris et al. (2022) using the constrained tree search option (Fig. 1).

In addition, each multiple sequence alignments were used to infer a maximum likelihood tree using IQ-Tree (Nguyen et al. 2015) using the Bayesian Information Criterion (BIC) to select the best fitting substitution model and empirical profile mixture models (C10–C60). A total of 1,000 ultrafast bootstrap replicates were used. These 216 gene trees were then summarized using ASTRAL (v2) (Mirarab et al. 2014). Importantly, this analysis recovers the same relationships among the 4 major bryophytes lineages as Fig. 1, specifically a monophyletic group of bryophytes that is sister to the land plants (Figure S1).

### Identifying novel and lost gene groups

The OrthoFinder gene count table (Orthogroups.GeneCount.tsv) was analyzed to identify the origin and loss of orthologous groups of proteins (OGs) based upon their taxonomic occupancy (Data S3). Importantly, these are made in the absence of phylogeny, so are robust to alternative evolutionary frameworks. Different sets of OGs can be analyzed (initially defined in Paps and Holland 2018):

- Novel Core (orthogroups present in all members of a clade and absent in all outgroup taxa). A conservative “all-present” criterion was adopted to mitigate the risk of false positives. Although this approach likely underestimates the total lineage-specific repertoire, it guarantees that the reported orthogroups are unique to the clade.
- Lost (orthogroups lost in the last common ancestor of a clade).

### Identifying duplicated and lost genes within orthogroups

To understand the role of gene duplication and subfamily loss in the diversification of bryophytes, gene tree–species tree reconciliation was used. Protein sequences of viable gene families (those with 4 or more members) were aligned with MAFFT (Katoh and Standley 2013) using auto method option. Aligned sequences were trimmed with TRIMAL (Capella-Gutiérrez et al. 2009) using gap threshold of 0.2, due to the large size of gene families. Maximum likelihood trees were inferred with IQTREE (Nguyen et al. 2015) as above. Additionally, 1,000 ultrafast bootstrap replicates were specified to estimate uncertainty as well as the option to write these to ufboot file with branch lengths. These ufboot tree files were used as input into AleRax (v.1.0.0) (Morel et al. 2024) to infer ancestral gene content and instances of gene duplication and loss. These inferences were based on the constrained species tree, which agree with the independent phylogenetic analysis recovering bryophyte monophyly. Results were visualized in R (v.3.4.2) (R Core Team 2014) using packages tidyr (Wickham and Henry 2018) and GGplot2 (Wickham 2016). Trees were plotted in iToL (Letunic and Bork 2019).

To obtain a functional description, proteins were assessed using Gene Ontology (Mi et al. 2017).

### Identifying xylem development genes

The literature was searched to identify genes involved in developmental pathways of vascular tissue. Genes from Ruonala et al. (2017), Sun et al. (2022), Nakano et al. (2015), Agusti and Blázquez (2020), Coll et al. (2011), Van Hautegem et al. (2015), Ramachandran et al. (2017), Xue et al. (2025), Du et al. (2024), Wang (2024), Mueller et al. (2025), Singla-Rastogi (2025), and Seo et al. (2020) were used (Data S4). To refine the evolutionary origins of these candidates, these genes were mapped to the expanded orthofinder dataset and reassessed their presence/absence patterns across a broader phylogenetic framework. Like previous studies (Bowles et al. 2022), many of these orthologs were estimated to predate the origin of land plants. However, by integrating improved taxon sampling—specifically from hornworts, lycophytes, ferns, and algal outgroups—the point of emergence for each orthogroup could be tracked. This allowed more precise pinpointing of whether these were ancestral innovations or later lineage-specific diversifications.

Analysis of vascular-related orthologs revealed broad conservation across land plants (Data S4). Among these, the NAC domain transcription factor family emerged as a primary candidate for further investigation, as they act as master regulators of xylem differentiation. To resolve the specific expansion and diversification history of this family, a targeted phylogenetic analysis was performed. For ease of visualization, a single representative species for each taxonomic group was chosen to produce a reduced phylogenetic tree, using the methods described above.

### Ancestral state reconstruction

The presence and absence of water-conducting cells was established from the published literature (Data S5). Two methodological approaches were used to estimate their evolutionary history considering the uncertain presence of these cells in liverworts. The tree topology was fixed based on the focal maximum likelihood analysis (Fig. 1). Ancestral states were estimated in R (R Core Team 2023) using the package phytools (Revell 2012). Briefly, likelihood ancestral states for discretely valued traits (eg presence/absence of water-conducting cells) were estimated using a continuous-time Markov chain model (Revell 2012). The MCMC approach is used to sample character histories from their posterior probability distribution (Huelsenbeck et al. 2003). To sample a greater portion of the character history, 1,000 stochastic maps were produced and summarized (Figure S2).

This analysis was repeated under the central phylogenetic hypothesis: bryophyte monophyly and alternative hypotheses for the placement of hornworts: (i) sister to vascular plants; and (ii) sister to all other land plants (Figures S3 and S4).

### Clustering of gene content

To understand genome diversity during early bryophyte evolution, count data for protein coding genes were clustered. Count data were transformed to presence and absence, and NMDS coordinates were calculated in R with Vegan and plotted with GGplot2 (Mirarab et al. 2014; Wickham 2016) (Figure S5).

## Results

### Gene loss and absence during bryophyte evolution

**Figure 2:**
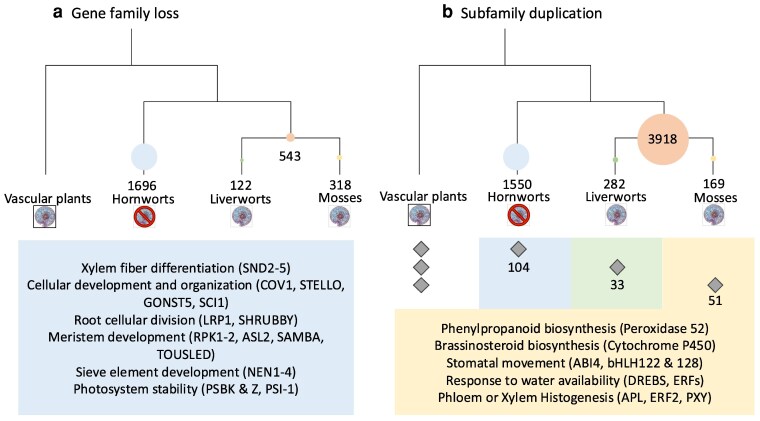
Gene loss and duplication within bryophytes. a) Gene loss in hornworts, liverworts, mosses, and setaphytes. The function of lost genes in hornworts is highlighted. b) Subfamily duplication in hornworts, liverworts, mosses, and setaphytes. The number of duplicates shared between vascular plants and each of hornworts, liverworts, and mosses is noted. The function of duplicated genes in mosses is highlighted.

Based on the inferences of a monophyletic group of bryophytes, analysis of ancestral gene content suggests that hornworts are the most genomically reduced bryophyte lineage (Fig. 2a). In total, 1,696 gene families were absent in the lineage leading to hornworts, compared with 122 and 318 in liverworts and mosses respectively. Based on the inferred monophyletic relationships of bryophytes, these gene families are lost with the origin of these lineages. Due to previous conflicting signals in bryophyte relationships, gene occupancy in pairs of bryophytes were inspected to understand whether reductive patterns of genome evolution have contributed to this uncertainty (Data S3). The sister lineages of liverworts and mosses lost 543 gene families compared to their common ancestor, hornworts and liverworts lost 775, and hornworts and mosses lost 4,284. This pattern is further confirmed with ordination analysis of gene content that demonstrates the proximity of mosses and liverworts to lycophytes and ferns in the theoretical gene space (Figure S5).

Several genes involved in vascular tissue development are absent in hornworts, accompanying the absence of water- and food-conducting cells in this group (Fig. 2a, Data S6). One of these lost gene groups SND2–5 (SECONDARY NAC DOMAIN Transcription Factors) is involved in xylem development where it regulates secondary cell wall development, downstream transcription factors, and programmed cell death (Zhong et al. 2021; Wei and Wei 2024). Bryophytes are not known to have secondary cell walls and therefore these genes may function in some other developmental process in mosses and liverworts. Indeed, SND2 is known to regulate the vascular tissue patterning processes of sieve elements, in coordination with SHR (SHORT ROOT) and NARS (NAC-REGULATED SEED MORPHOLOGY 1) (Kim et al. 2020). Also lost in hornworts is ASYMMETRIC LEAVES 1–2 (also known as PHANTASTICA). This is involved in leaf vasculature development by positive regulation of HD-ZIP III transcription factors REV (REVOLUTA), PHV (PHAVOLUTA), and PHB (PHABULOSA) (Ramachandran et al. 2017). Another set of lost genes NEN1–4 (NAC45/86-DEPENDENT EXONUCLEASE-DOMAIN PROTEIN 1–4) promote sieve element development, particularly the later stages of differentiation (Roszak et al. 2021). Additionally lost in hornworts was SHRUBBY, known to function in radial patterning of root meristems. This is regulated through interaction with the SHR, SCR (SCARECROW), and PLT (PLETHORA) pathway known to be involved with root vascular patterning (Koizumi and Gallagher 2013; Ramachandran et al. 2017). The SHI gene family has also been lost in hornworts, known to regulate growth and development (Fang et al. 2023). Other genes lost in hornworts known to be involved in development include Style cell-Cycle Inhibitor 1, TOUSLED, RPK1–2, LUMINIDEPENDENS, and SAMBA. Several genes were also linked to losses in photosynthetic function (PSI-1, Photosystem II reaction center protein H/N/Z, psbK) which accompanies a previously identified reversion back to a monoplastidic phenotype in hornworts (MacLeod et al. 2022). This points to extensive developmental gene loss coinciding with the absence of water-conducting cells in hornworts.

The above losses were defined by presence and absence of genes in specific bryophyte lineages, regardless of inferred phylogeny. Given the complex nature of plant genome evolution, gene loss can also be detected within orthogroups, manifesting as subfamily losses. Analysis of subfamily dynamics, based on the inferred monophyletic relationship of bryophytes, reveals substantial losses in hornworts and mosses (Data S7). These patterns of reductive evolution in hornworts and mosses were 2-fold higher than liverworts. Consistent with previous analyses using genome data (Harris et al. 2022), bryophytes were characterized by large rates of gene loss (Data S7). This supports the role of loss as well as gene gain in shaping the evolution of bryophytes.

### Bryophyte evolution by gene duplication

Comparison of individual gene trees with the inferred monophyletic bryophyte species tree identified hornworts had the highest rate of subfamily duplication (1,550 orthogroups) (Fig. 2b). Liverworts and mosses exhibited lower rates of gene duplication with 282 and 169 respectively. Hornworts also exhibited the highest rate of convergent gene duplication with vascular plants estimated at 104 orthogroups (Fig. 2b, Data S7). By contrast, mosses have 51 convergently duplicated orthogroups while liverworts have 33 of these.

Considering that approximately one-third of moss duplicate genes also diversified in vascular plants, the functions of these genes were investigated (Fig. 2b, Data S8 and S9) using PantherGO (Mi et al. 2017). Several were related to metabolism and secondary metabolite biosynthesis, including phenylpropanoids (MYBs, Peroxidase 52) and hormone biosynthesis pathways such as gibberellins and brassinosteroids (gibberellin β-dioxygenases, cytochrome P450s). Signal transduction and responses to environmental stressors were also common, including responses to water availability (DEHYDRATION RESPONSIVE ELEMENT BINDING proteins [DREBs], ETHYLENE RESPONSE FACTORS ERFs]) and gravity (Tubulin alpha-3/4/6 chains), as well as stomatal movement (ABA-INSENSITIVE 4 [ABI4], bHLH122, and bHLH128). Convergent gene duplications between mosses and vascular plants were also associated with growth and development (Data S8 and S9). Terms related to cell wall biogenesis (xyloglucan endotransglucosylases), cell wall organization and modification (expansins, peroxidases), and plant organ development (AGAMOUS-LIKE genes [AGLs], MADS-box genes) were also associated.

Among these developmental categories were genes linked to phloem and xylem histogenesis, the process of tissue formation from vascular precursor cells, including APL (ALTERED PHLOEM DEVELOPMENT), ERF2 (ETHYLENE RESPONSE FACTOR 2), and PXY (PHLOEM INTERCALATED WITH XYLEM). Together, these patterns of extensive gene loss in hornworts, which lack water-conducting tissues, and convergent gene duplication between water-conducting mosses and tracheophytes motivated further investigation into the evolutionary genomics of vascular tissue.

### The evolutionary genetics of vascular tissue development

**Figure 3:**
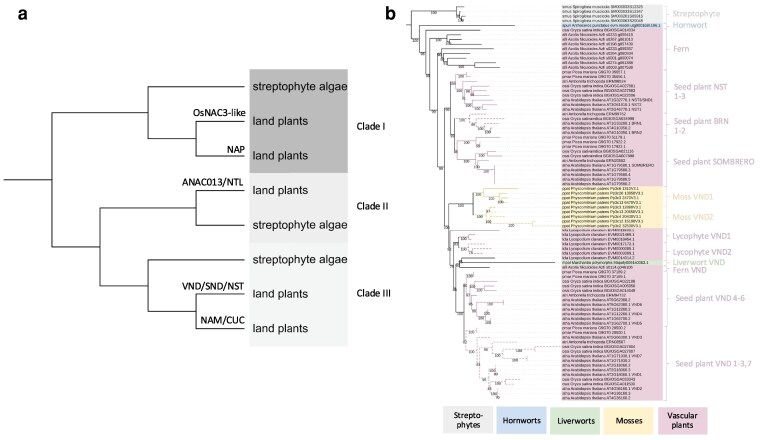
Evolution and diversification of vascular NAC domain (VND) transcription factors in land plants. a) Summarized phylogeny of the NAC transcription factor family. The shaded regions highlight the 3 primary evolutionary clades. Abbreviations stand for OsNac-like (*Oryza sativa* NAC-like), NAP (NAC-like, activated by AP3/PI), ANAC013/NTL (*Arabidopsis* NAC domain-containing protein 13/NAC with Transmembrane Motif-like), and NAM/CUC (NO APICAL MERISTEM/CUP-SHAPED COTYLEDON). b) Detailed phylogenetic tree of the VND subfamily. A representative species for each taxonomic group was chosen to produce a reduced phylogenetic tree. Colored background blocks denote major plant lineages. Specific sub-clades are labeled based on functional orthologs in *Arabidopsis thaliana*, including NST (NAC SECONDARY WALL THICKENING PROMOTING FACTOR), BRN (BEARSKIN), and SOMBRERO.

The ancestor of vascular plants had the same number of key developmental regulators as the ancestor of land plants, regardless of the inferred embryophyte relationships (Fig. 3, Data S4). VASCULAR-RELATED NAC DOMAIN (VND) transcription factors, a key regulator of xylem development (Endo et al. 2015), emerged in the ancestor of Phragmoplastophyta, which subsequently diversified into 3 streptophyte groups. One group duplicated in the land plant ancestor, leading to VND1–7 (Fig. 3a). Though originating in land plants, these 7 genes diversified within seed plants, implying there was a single ancestral copy in the first tracheophyte and embryophyte (Fig. 3). Importantly this has been shown to have functional equivalence across land plant evolution (Xu et al. 2014). Importantly, this gene appears to have been lost in hornworts, coinciding with the absence of these cells. NAC transcription factors involved in secondary cell wall development are specific to seed plants, which exhibit complex vascular morphology (Fig. 3) (Mitsuda et al. 2007; Bennett et al. 2010). A subgroup containing SND1, NST1–2 (NAC SECONDARY WALL THICKENING PROMOTING FACTOR1), BEARSKIN1–2, and SOMBRERO, are specific to seed plants and diversify through duplication (Fig. 3b). This suggests the first tracheophytes developed vascular tissue without these genes.

**Figure 4:**
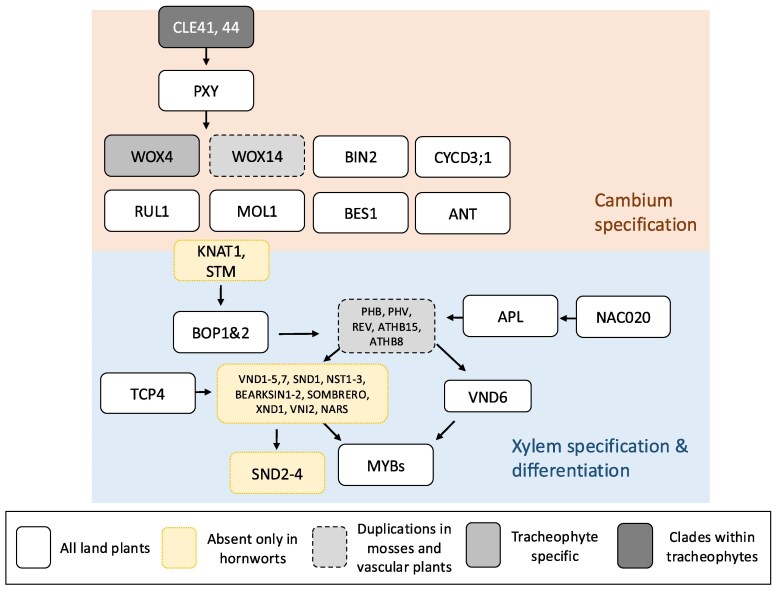
The gradual evolution of genetic pathways. The evolutionary trajectory of the plant vascular development pathway. Schematic representation illustrating the transition from ancient land plant regulators to specialized pathways for cambium specification and xylem differentiation. Labels at the bottom indicate key evolutionary events.

Hornwort-specific losses, based on the inferred monophyly of bryophytes, were also identified in gene families acting both upstream and downstream of VNDs, specifically STM (SHOOT MERISTEMLESS) and SND2–4 (Fig. 4, Data S4). As previously mentioned, SND2–4 function downstream of VND regulators to control vascular tissue patterning and secondary cell wall formation, while STM maintains meristematic identity in the shoot apical meristem, providing the developmental context from which vascular tissues are specified (Scofield et al. 2013). A subfamily containing xylogen and xylogen-like arabinogalactan proteins also appears to be absent in hornworts (Data S4). The concurrent loss of these key families suggests that hornworts lack much of the conserved genetic network underlying xylem differentiation and water-conducting cell specialization in other land plants, consistent with the absence of these cells.

Several developmental genes duplicate convergently across tracheophytes and water-conducting mosses, regardless of the inferred bryophyte relationships (Fig. 4, Data S4). Amongst these, REVOLUTA and PHABULOSA are involved in xylem specification (Růžička et al. 2015), while LOGs activate key biosynthesis genes which promote cell division in the neighboring cells (Růžička et al. 2015). WOXs (WUSCHEL-RELATED HOMEOBOXs) regulate the maintenance of procambial/cambial cells (Yasui et al. 2018). Their independent duplication in tracheophytes and mosses could potentially point to a molecular signature of convergent evolution. Finally, several genes (eg LHW) have tracheophyte-specific patterns of diversification, pointing to novelty within vascular plants (Data S4). These include xylogen and xylogen-like arabinogalactan proteins important for the continuous, coordinated development and morphogenesis of the plant vascular system (Ruonala et al. 2017). Additionally, WOX4 also duplicates in the ancestor of land plants, but one clade is lost in all bryophyte lineages, consistent with previous studies (Harris et al. 2022).

Together, these 3 categories of genes, those conserved across water-conducting plants, those convergently evolving in tracheophytes and water-conducting mosses and those exhibiting tracheophyte-specific novelty, suggests that rather than a sudden emergence, water-conducting tissues have evolved gradually in a piecemeal fashion, building upon existing genetic mechanisms.

### Ancestral states of water conduction

Ancestral state reconstruction revealed a range of possible ancestral states in the last common ancestor of bryophytes and embryophytes, based on the inferred monophyly of bryophytes (Figure S2). To incorporate uncertainty about their presence in liverworts, 2 reconstructions were completed, 1 where the uncertainty of water-conducting tissues in liverworts were considered (Figure S2a) and 1 where only liverworts with clear evidence of water-conducting cells are included (Figure S2b). In this analysis, water-conducting tissues were considered as present or absent rather than dissecting them further, such that the potential for homology was incorporated. In the first analysis, water-conducting tissues were predicted in the ancestor of bryophytes and embryophytes (Figure S2a). In the latter, water-conducting tissues were absent from the ancestral bryophytes and embryophytes (Figure S2b). Instead, these appeared separately in vascular plants, within liverworts, and within mosses.

To consider bryophyte paraphyly, additional analyses were conducted (Data S10, Figures S3 and S4), altering the placement of hornworts as sister to vascular plants and sister to land plants respectively. Considering hornworts as sister group to vascular plants, water conduction is absent in land plants under both scenarios (Figure S3). Under hornworts as sister group to all other land plants, these inferences are the same (Figure S4).

## Discussion

Overall, these analyses provide novel insights into the molecular evolution of bryophytes and the earliest land plants. Through integration of these inferences, the evolutionary history of developmental genes associated with vascular tissues and water-conducting cells can be reconstructed. Notably, the results highlight that bryophytes represent a genomically diverse group, reflecting lineage-specific retention, gene loss, and innovation.

Phylogenetic analysis of 216 genes using ASTRAL inferred a fully resolved land plant phylogeny (Fig. 1). Notably, bryophytes were resolved as a monophyletic group (Figure S1), with full bootstrap support for bryophytes, mosses, liverworts, and hornworts. This topology is consistent with numerous recent phylogenomic studies (de Sousa et al. 2019; Leebens-Mack et al. 2019; Li et al. 2020; Su et al. 2021; Harris et al. 2022; Healey et al. 2023; Hu et al. 2023; Li et al. 2024; Bowles et al. 2024a, 2024b; Chen and de Vries 2025; Dong et al. 2025; Shen et al. 2025; Xue et al. 2026). This provides an evolutionary framework for interpreting patterns of genome evolution and reconstructing the origins of water-conducting tissues and other key innovations in terrestrial plants.

The work presented here adds to the growing body showing that bryophytes are a genomically diverse and evolutionarily innovative assemblage (Fig. 2). Based on the inferred monophyletic relationship of bryophytes, these suggest the last common ancestor of land plants may have been genomically complex (Fig. 2), already possessing a rich set of developmental and stress–response genes. The bryophytes subsequently underwent their own distinct evolutionary trajectory involving extensive gene loss and simplification (Harris et al. 2022). A scan of 123 recently sequenced bryophyte genomes demonstrated that despite their modest individual sizes, they collectively retain a broader and more diverse gene family repertoire than tracheophytes, reflecting widespread lineage-specific innovation and retention (Dong et al. 2025). Complementing this view, rounds of ancient genome wide duplication events within early bryophytes have been identified, that likely facilitated adaptation to terrestrial environments through neofunctionalization of duplicated genes (Shen et al. 2025). In addition, genome size shows contrasting paths within bryophyte lineages; mosses underwent repeated duplication and downsizing, liverworts maintained chromosomal stability and slower genomic change (Fujiwara et al. 2025). Combined with the analysis presented here, this reveals bryophytes as descendants of a complex ancestor rather than static relics of primitive forms. This underscores that bryophytes are not basal, but rather an evolutionarily dynamic clade (McDaniel 2021).

Gene loss and gene family evolution can be inferred using multiple approaches, which could lead to discrepancies between studies (Harris et al. 2022; Dong et al. 2025). Orthogroup-based methods (eg Count) detect losses by the absence of homologous genes in modern genomes, but are sensitive to incomplete assemblies, annotation errors, or high sequence divergence (Bowles et al. 2020). Phylogenetic reconciliation methods (eg, AleRax) model gene duplication and loss along a species tree, estimating ancestral gene content, but rely on accurate gene trees and assumptions about duplication/loss rates (Morel et al. 2024). These differences in approach could therefore lead to different reconstructions of ancestral gene content. The analysis presented here identifying genome simplification in hornworts is consistent across methods, supporting robust evolutionary conclusions (Fig. 2). In addition, one of these methods (AleRax) is based on an inferred monophyletic relationship of bryophytes while the other is agnostic to phylogeny (Orthogroup-based methods), providing support to hornwort gene loss.

Based on the inferred monophyletic relationship of bryophytes, the finding that key developmental regulators of vascular differentiation were already present in the ancestor of land plants indicates that the molecular foundations for vascular differentiation long predate the emergence of lignified conducting tissues (Fig. 3). This supports the hypothesis that the genetic architecture underlying water transport systems was progressively assembled and modified, rather than arising de novo with tracheophytes. Such a scenario aligns with the proposed deep and gradual origin of transporting tissues across land plants (Woudenberg et al. 2022), in which innovations in water conduction reflect a continuum of functional diversification rather than discrete evolutionary leaps. The persistence of water-conducting cells in many moss lineages (Brodribb et al. 2020) and their physiological parallels to xylem tracheids reinforces this model, suggesting that ancestral regulatory networks were repeatedly co-opted and refined during early terrestrial evolution.

The emergence of NAC domain transcription factors in the Phragmoplastophyta and their subsequent duplication in early land plants suggest that the ancestral regulatory toolkit was co-opted for xylem specification, preceding the diversification of specialized NAC genes (eg, SND1, NST1/2) in seed plants (Fig. 3). The evolutionary trajectory of VNDs, marked by their early origin and later subfunctionalization, is consistent with the stepwise diversification of developmental networks (Mitsuda et al. 2007; Endo et al. 2015; Kim et al. 2025). Functional studies have demonstrated that NAC domain regulators maintain conserved roles across bryophytes and tracheophytes (Xu et al. 2014), supporting the interpretation that these transcription factors represent a deeply conserved module that was co-opted for vascular development. Seed plant specific expansions of secondary wall-associated NACs (eg, SND1, NST1/2, BEARSKIN1/2, SOMBRERO) likely reflects diversification associated with the rise of more complex vascular morphologies. With the diversification of the VND gene family occurring within seed plants (Fig. 3), this raises an important question. Rather than reflecting the ancestral vascular developmental toolkit, much of what is understood to be vascular plant specific may in fact be seed plant specific. This phenomenon has arisen due to the reliance on flowering and seed plant model systems (Woudenberg et al. 2022). Analysis of VND gene function in lycophytes and ferns is required to fully understand their role in the evolutionary trajectory of water-conducting tissues.

Parallel diversification of REVOLUTA and PHABULOSA (Fig. 4), which mediate xylem specification, alongside LOG genes involved in cytokinin biosynthesis (Data S4), further highlights how multiple regulatory pathways independently diversified in tracheophytes and mosses with conducting cells (Růžička et al. 2015). The independent evolution of WOX genes in mosses and vascular plants (Fig. 4) suggests repeated recruitment of an ancient stem-cell regulatory system for tissue patterning (Jha et al. 2020). These patterns of parallel diversification and functional convergence are consistent with a mosaic of evolutionary mechanisms, in which ancestral regulators were redeployed in distinct lineages to generate similar developmental outcomes (Bowles et al. 2022).

Taken together, these results suggest that the evolution of water-conducting tissues was not characterized by a sudden innovation but rather by a piecemeal, iterative assembly of preexisting genetic mechanisms (Fig. 4). This interpretation echoes the broader principle that morphological novelties often arise through the reorganization of conserved genetic modules rather than the invention of entirely new genes (Woudenberg et al. 2022). Recent investigations into xylogen or xylogen-like arabinogalactan proteins (AGPs) in bryophyte genomes (Mueller et al. 2025) support the hypothesis that an evolutionary continuity is more likely than a strict gap between the water-conducting tissues of bryophytes and vascular plants. The retention of homologous regulators across bryophytes and tracheophytes highlights the continuity of developmental control throughout land plant evolution, while their lineage-specific diversification demonstrates how incremental molecular changes can yield major morphological innovations.

The main ancestral state reconstruction analysis predicted water-conducting tissues were present in the common ancestor of bryophytes and embryophytes respectively (Figure S2). This finding is supported by both evolutionary parsimony and functional biophysical constraints. From a phylogenetic perspective, a single ancestral emergence followed by varying degrees of phenotypic sophistication—alongside a secondary loss in the hornwort lineage—represents a highly parsimonious evolutionary scenario for land plants. Furthermore, this model aligns with the physiological imperatives of a terrestrial existence (Woudenberg et al. 2022). Regardless of physical scale, a complex multicellular body plan operating within a subaerial habitat inherently necessitates the efficient distribution of water and solutes to combat desiccation (Sperry 2003).

These interpretations are made in the context of an inferred monophyletic bryophyte clade (Figs. 1–4, Data S2). While this topology is supported by many recent phylogenomic analyses, alternative hypotheses are considered. Following Puttick et al. (2018), which recovers a monophyletic setaphyte clade (liverworts + mosses), the alternative placements of hornworts is focused on: (i) within a monophyletic bryophyte clade (as above); (ii) as sister to vascular plants; and (iii) as sister to all other land plants. Under a hornwort–tracheophyte sister relationship, the interpretation of the genomic result is largely unchanged. Gene loss inferred from the orthogroup-based approach (which is agnostic to phylogeny) is still recovered along the hornwort lineage, consistent with the absence of water-conducting cells. Similarly, convergent gene duplication between vascular plants and water-conducting mosses is retained, as this inference is based on manual inspection of gene trees. Ancestral state reconstructions differ slightly, indicating independent origins of water conduction in liverworts, mosses, and vascular plants (Data S10, Figure S3).

Under the hornworts as sister to all other land plants scenario, the interpretation of gene loss would be altered slightly. Gene loss would instead be split into 2 categories: (i) for genes only found in land plants, these would reflect a novelty of the ancestral node of setaphytes and tracheophytes (920 orthogroups: Data S6); and (ii) for genes found in land plants and streptophyte algae, these would still remain as losses from hornworts (776 orthogroups: Data S6). Of the 13 gene families discussed in the text above, 11 remain as hornwort losses, including SND2–5, NEN1–4 and SHRUBBY (Data S6). Only ASYMMETRIC LEAVES 1–2 and RPK1–2 would be considered novelties of setaphytes and tracheophytes. The signal of convergent gene duplication between vascular plants and water-conducting mosses remains robust as this is based on manual inspection of gene trees. Ancestral state reconstructions in this scenario suggest either the presence of water-conducting traits in the common ancestor of setaphytes and tracheophytes or multiple independent origins, depending on how water-conducting tissues in liverworts are defined (Data S10, Figure S4).

This work is based on the inferences that bryophytes are monophyletic (Figs. 1–4, Data S2). However, across all phylogenetic scenarios, several patterns are consistent. First, the signal of genome simplification in hornworts, inferred from a phylogeny-agnostic orthogroup-based approach, is robust to topological uncertainty (Fig. 2). Second, convergent gene duplication in vascular plants and water-conducting mosses is consistently recovered (Fig. 4). Third, certain genes (eg LHW) show tracheophyte diversification, indicating these are specific innovations of vascular plants (Fig. 4). Collectively, these results support a model in which heterogeneous evolutionary mechanisms—including gene loss, convergent duplication, and tracheophyte-specific diversification—contributed to the evolution of water-conducting tissues.

Future work integrating single-cell transcriptomics and functional genomics across bryophyte and tracheophyte representatives will be crucial for resolving how genes are regulated at the cellular level. Experimental characterization of VND, WOX, and NAC orthologs in nonvascular taxa could reveal whether their ancestral roles were linked to simple water transport or broader stress-related functions that later became canalized into vascular differentiation. Such insights will further refine our understanding of how the genetic foundations for one of the most transformative plant innovations—the vascular system—were assembled gradually from an ancient toolkit. Overall, these analyses shed new light on the genomic foundations of bryophyte evolution, revealing how gradual and modular changes in ancient regulatory networks underpinned one of the most consequential transitions in Earth’s history, the diversification of plant life on land.

## Supplementary Material

kiag525_Supplementary_Data

## Data Availability

The input data underlying this article are available in Data S1. The output gene trees are available at https://itol.embl.de/shared/Bowles_et_al and the code has been uploaded to https://github.com/alexmcbowles/Bowles_et_al_2026.
