# Supplementary material for: Phylogenomic analysis supports the deep and gradual evolution of water-conducting tissues
Source: Plant Physiol. 2026 Jul 18;201(4):kiag525. doi: 10.1093/plphys/kiag525 (PMC13464504; doi:10.1093/plphys/kiag525)
Supplement: Supplementary file 1 [file kiag525_supplementary_data.zip › Supplementary Information.docx]

**Supplementary Information**

**Supplementary Figure 1:** Phylogenetic tree of land plants based on ASTRAL analysis of 216 single copy orthogroups, inferring a monophyletic group of bryophytes. Bootstraps highlight support at each node. Taxa are colour coded as follows; outgroup: grey, vascular plants: red, hornworts: blue, liverworts: green, mosses: yellow.

**Supplementary Figure 2:** Ancestral state reconstruction of water conducting tissues, based on the inferred phylogeny including monophyletic bryophytes. **A)** This reconstruction considers the uncertainty of water conducting tissues in liverworts. **B)** This reconstruction only considers liverworts where there is clear evidence of water conducting cells.

**Supplementary Figure 3:** Ancestral state reconstruction of water conducting tissues, based on phylogeny specifying hornworts as sister to vascular plants. **A)** This reconstruction considers the uncertainty of water conducting tissues in liverworts. **B)** This reconstruction only considers liverworts where there is clear evidence of water conducting cells.

**Supplementary Figure 4:** Ancestral state reconstruction of water conducting tissues, based on phylogeny specifying hornworts as sister to all other land plants. **A)** This reconstruction considers the uncertainty of water conducting tissues in liverworts. **B)** This reconstruction only considers liverworts where there is clear evidence of water conducting cells.

**Supplementary Figure 5:** Ordination analysis of gene space. Each point reflects an individual species, colour coded by its phylogenetic group. Polygon group major phylogenetic groups.
